# Activity of a trinuclear platinum complex in human ovarian cancer cell lines sensitive and resistant to cisplatin: cytotoxicity and induction and gene-specific repair of DNA lesions

**DOI:** 10.1054/bjoc.2001.1751

**Published:** 2001-05

**Authors:** G Colella, M Pennati, A Bearzatto, R Leone, D Colangelo, C Manzotti, M G Daidone, N Zaffaroni

**Affiliations:** Department of Experimental Oncology, Istituto Nazionale per lo Studio e la Cura dei Tumori, Via Venezian 1, 20133 Milan; Department of Pharmacology, Università degli Studi di Verona, Via delle Menegone 10, 37134 Verona; Department of Medical Sciences, Università degli Studi di Torino, Via Solaroli 7, 28100 Novara; Novuspharma S.p.A., Viale Stucchi 110,20052 Monza, Italy

**Keywords:** BBR 3464, cisplatin, DNA repair, ovarian cancer

## Abstract

A collateral sensitivity or a very modest cross-resistance to BBR 3464 was found in 2 ovarian cancer cell lines with experimentally induced resistance to cisplatin. Loss of mismatch repair proteins (hMLH1, hPMS2) or overexpression of nucleotide excision repair proteins (ERCC1) was not detrimental for the cellular sensitivity to BBR 3464. Moreover, interesting differences in the kinetics of formation and removal of DNA lesions at the single-gene (N- *ras*) level were observed between BBR 3464 and CDDP. © 2001 Cancer Research Campaign www.bjcancer.com


*British Journal of Cancer* (2001) **84**(10), 1387–1390
© 2001 Cancer Research Campaign
doi: 10.1054/ bjoc.2001.1751, available online at http://www.idealibrary.com on IDEAL®

http://www.bjcancer.com

Short communication

### Activity of a trinuclear platinum complex in human ovarian cancer cell lines sensitive and resistant to cisplatin: cytotoxicity and induction and gene-specific repair of DNA lesions

**G Colella[1], M Pennati[1], A Bearzatto[1], R Leone[2], D Colangelo[3], C Manzotti[4], MG Daidone[1] and N Zaffaroni[1]**

[1]Department of Experimental Oncology, Istituto Nazionale per lo Studio e la Cura dei Tumori, Via Venezian 1, 20133 Milan; [2]Department of Pharmacology, Università degli Studi di Verona, Via delle Menegone 10, 37134 Verona; [3]Department of Medical Sciences, Università degli Studi di Torino, Via Solaroli 7, 28100 Novara; [4]Novuspharma S.p.A., Viale Stucchi 110,20052 Monza, Italy

**Summary** A collateral sensitivity or a very modest cross-resistance to BBR 3464 was found in 2 ovarian cancer cell lines with experimentally induced resistance to cisplatin. Loss of mismatch repair proteins (hMLH1, hPMS2) or overexpression of nucleotide excision repair proteins (ERCC1) was not detrimental for the cellular sensitivity to BBR 3464. Moreover, interesting differences in the kinetics of formation and removal of DNA lesions at the single-gene (N-*ras*) level were observed between BBR 3464 and CDDP. © 2001 Cancer Research Campaign http://www.bjcancer.com

**Keywords**: BBR 3464; cisplatin; DNA repair; ovarian cancer

Cisplatin (CDDP) is one of the most effective drugs for the treatment of human solid tumours. However, its therapeutic efficacy is limited by the emergence of tumour cell subpopulations with intrinsic or treatment-induced resistance. Multinuclear platinum compounds represent a new approach to circumvent cellular resistance to CDDP in that they are endowed with a different DNA-binding profile compared to their mononuclear counterparts (Perego et al, 1999; Manzotti et al, 2000). BBR 3464, which has been identified as the most active member of this class of compounds, is more potent than CDDP (Brabec et al, 1999) and is active in CDDP-resistant experimental human tumour models (Pratesi et al, 1999). However, the cellular determinants responsible for BBR 3464 activity are largely unknown.

In the present study we evaluated the cytotoxic activity of BBR 3464 in 2 pairs of human ovarian cancer cell lines sensitive and with experimentally induced resistance to CDDP. Moreover, in an attempt to elucidate the relevance of DNA repair on the cellular response to BBR 3464, we assessed in our cellular models the expression of mismatch repair (MMR) and nucleotide excision repair (NER) genes as well as the kinetics of accumulation and removal of drug-induced lesions at the single-gene (N-*ras*) level.

### MATERIALS AND METHODS

### Cell lines and growth inhibition assay

Two human ovarian cancer cell lines, OAW42 and A2780, and their corresponding CDDP-resistant sublines. OAW42MER (Britten et al, 1990) and A2780cp8 (Beherens et al, 1987) were used in the study. The A2780d cell clone, which was selected in culture without drug exposure was also used. Cells in the logarithmic growth phase were exposed to various concentrations of CDDP (Pharmacia-Upjohn, Milan, Italy) and BBR 3464 (Novuspharma S.p.A., Monza, Italy) for 1 h. After a 72-h incubation with drug-free medium, cells were trypsinized and counted in a particle counter (Coulter Counter, Coulter Electronics, Luton, UK). The results were expressed as the number of cells in treated samples compared with control samples. In vitro drug activity was expressed as the concentration able to inhibit cell growth in treated samples by 50% ($IC_{50}$).

*Received 25 October 2000*
*Revised 13 February 2001*
*Accepted 16 February 2001*

*Correspondence to:* N Zaffaroni

### Nuclear extracts and western blotting

Nuclear lysate (Dignam et al, 1983) was separated on 8% or 12% SDS-PAGE and transferred to nitrocellulose. Filters were probed with the primary antibodies anti-hMLH1, anti-hMSH2, anti-hPMS2 (Santa Cruz Biotechnology, Santa Cruz, CA, USA), anti-ERCC1 and anti-tubulin β (Neomarkers, Union City, CA, USA), and then incubated with the secondary anti-mouse or anti-rabbit IgG horseradish peroxidase-linked whole antibodies (Amersham Pharmacia Biotech, Uppsala, Sweden). Bound antibody was detected using the enhanced chemoluminescence Western blotting detection system (Amersham).

### RNA isolation and Reverse Transcription-PCR

Total RNA was extracted from frozen cells using Trizol reagent (Life Technologies Italia, Milan, Italy). Reverse transcription-PCR was performed by means of the RNA PCR Core Kit (Perkin Elmer-Roche, Foster City, CA, USA). Primers used were: ERCC1 sense 5′-GTGCAGTCGGCCAGGATACAC-3′ and antisense 5′-GTCCTCCTGGAGTGGCCAAG-3′, β-actin sense 5′-GGGAATTCAAAACTGGAACGGTGAAGG-3′ and antisense 5′-GGAAGCTTATCAAAGTCCTCGGCCACA-3′. PCR products were loaded on a 3% agarose gel and bands were quantified after staining with ethidium bromide.

### Measurement of platinum DNA lesions by quantitative PCR

Cells were incubated for 5 h with CDDP and BBR 3464 and collected immediately after drug removal or following an additional 6,24 or 48 h of incubation in drug-free medium. Cell monolayers were recovered from the Petri dish using a cell scraper, pelleted by centrifugation and counted. The cell pellet was resuspended in lysis buffer and incubated at 60˚C for 2 h and at 95˚C for 15 min. After centrifugation the DNA-containing cell lysate was diluted and the quantitative PCR assay was performed as described (Koberle et al, 1996) by means of N-*ras* sense (5′-CCTTAATCTGTCCAAAGCAGAGGC-3′) and antisense (5′-CAGCAAGAACCTGTTGGAAACCAG-3′) primers, and β-actin sense and antisense primers. PCR products were separated on a 5% non-denaturing polyacrylamide gel and visualized by autoradiography. Quantification of PCR products was performed by densitometric analysis using ImageQuant software (Microsoft Corp., Santa Rosa, CA, USA).

### RESULTS AND DISCUSSION

Results of cytotoxicity experiments indicated a collateral sensitivity of OAW42MER cells to BBR 3464. In fact, whereas the cells were 10 times less sensitive to CDDP than the parental OAW42 cells ($IC_{50}$: 83.0 ± 18.6 μM vs 8.3 ± 2.8 μM), they were 14 times more sensitive to BBR 3464 ($IC_{50}$: 0.36 ± 0.14 μM vs 5.20 ± 1.3 μM). As regards the second pair of cell lines. A2780cp8 cells were 14-fold more resistant to CDDP ($IC_{50}$: 60.0 ± 5.6 μM vs 4.3 ± 0.35 μM) and only 2.5-fold more resistant to BBR 3464 ($IC_{50}$: 0.20 ± 0.095 μM vs 0.08 ± 0.008 μM) than the parental A2780 cells.

OAW42MER and A2780cp8 cells lacked the expression of the MMR proteins hMLH1 and hPMS2 (Figure 1A) and, compared to the MMR-proficient OAW42 and A2780 parental cell lines, showed a marked instability of the microsatellite sequences at several loci (data not shown). Superimposable results were observed in the A2780d cell clone, which was selected in culture without drug exposure and showed a 4.7-fold resistance to CDDP ($IC_{50}$ : 20.0 ± 3.25 μM) and exactly the same sensitivity to BBR 3464 ($IC_{50}$ : 0.077 ± 0.03 μM) as the parental A2780 cell line, which suggests, in agreement with previous findings (Perego et al, 1999), a lack of influence of MMR status on BBR 3464 cytotoxic activity. As regards the NER system. ERCC1 mRNA and protein levels of the OAW42MER cell line were 3 times higher than those of the OAW42 parental cell line, whereas a comparable expression of the NER gene was found in the A2780, A2780d and A2780cp8 cells (Figure 1B,C). It has been reported that in Chinese hamster ovarian cells overexpression of ERCC1 may enhance the sensitivity to alkylating agents because a greater number of DNA lesions are induced than the other enzymes involved in the pathway can repair (Bramson and Panasci, 1993). This could partially explain why among our cell lines those with a high level of ERCC1 (OAW42MER, A2780 and A2780cp8) were more sensitive to BBR 3464 than the OAW42 cells, which express less protein. In fact, the trinuclear compound can produce bulky DNA lesions, thus inducing ERCC1 to make more incisions than the remaining NER proteins are able to repair.

Results obtained from atomic absorption spectrometry and mass spectroscopy analysis, following incubation of cells with equimolar concentrations of the 2 drugs, indicated that the intra-

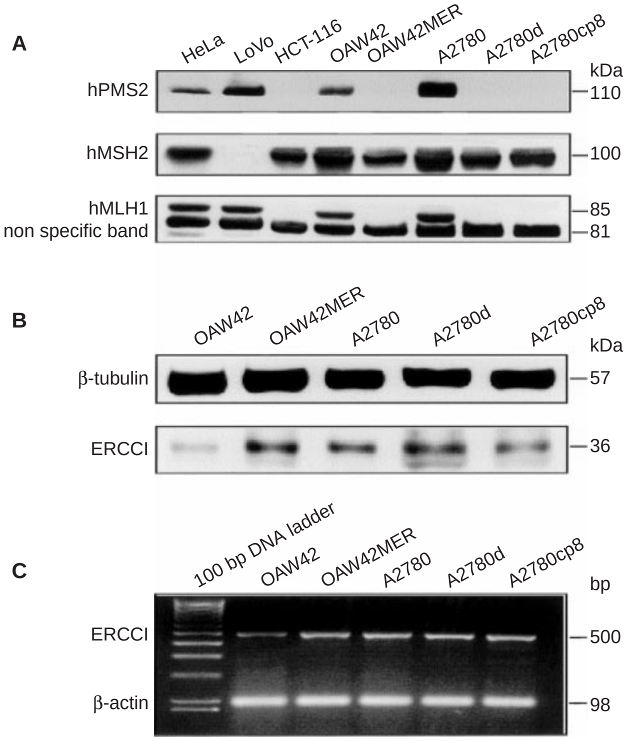

**Figure 1** Analysis of DNA mismatch repair (MMR) and nucleotide excision repair (NER) status in ovarian cancer cell lines. (**A**) Western blotting analysis of hPMS2, hMSH2 and hMLH1 protein expression. HeLa, LoVo and HCT-116 cell lines were used as specific controls for MMR protein expression. The non-specific lower band in the hMLH1 blot represented a control of protein loading. (**B**) Western blotting analysis of ERCC1 protein. (**C**) RT-PCR analysis of ERCC1 mRNA

cellular accumulation of platinum as well as the extent of platinum bound to DNA was always higher after treatment with BBR 3464 than with CDDP. Such a finding is consistent with an increased affinity of BBR 3464 to DNA owing to the presence of 2 reactive platinum centres in the molecule and to the high positive charge (+4) which could contribute to drug interaction with DNA through electrostatic and hydrogen binding (Brabec et al, 1999). However, whereas in CDDP-resistant cells the levels of intracellular platinum and DNA platination were consistently lower than those detected in sensitive cells, no differences in these parameters among cell lines, able to justify their peculiar sensitivity/resistance profile to BBR 3464, were observed (data not shown).

We did not perform any evaluation on the efficiency of detoxification systems (in terms of glutathione and metallothionein expression) in our cell lines and, therefore, we do not know whether the detoxification process influences the cellular response to BBR 3464.

In an attempt to identify possible molecular determinants of cellular response to BBR 3464, we focused on the kinetics of gene-specific repair of lesions produced by the trinuclear platinum complex in genomic DNA as assessed by a quantitative PCR on the 523 bp sequence of the N-*ras* gene intron I. A 98 bp β-actin gene fragment was coamplified to normalize experimental variability among samples. Results of preliminary experiments performed using as a template naked genomic DNA exposed in vitro to BBR 3464 and CDDP, showed that the extent of amplification inhibition and, as a consequence, the level of lesions present in the N-*ras* gene were dependent on drug concentration for both

 © 2001 Cancer Research Campaign

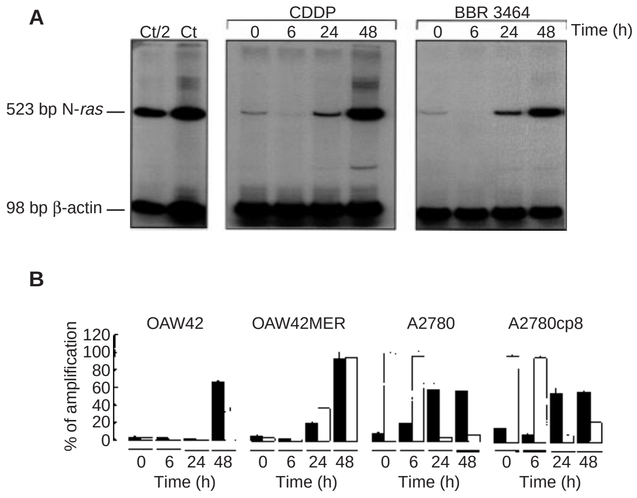

**Figure 2** **(A)** A representative example of quantitative PCR analysis on genomic DNA (N-*ras* gene) after a 5-h drug exposure of OAW42MER cells to the $IC_{50}$ of CDDP and 10-fold $IC_{50}$ of BBR 3464 and after an additional 6,24 or 48 h of incubation in drug-free medium. Controls: Ct/2 = 625 cell equivalents. Ct = 1250 cell equivalents. (**B**) Quantification of PCR reactions for the N-*ras* gene following a 5-h incubation to $IC_{50}$ of CDDP (■) and $10 \times IC_{50}$ of BBR 3464 (□) in OAW42, OAW42MER, A2780 and A2780cp8 cell lines. The ordinate shows the relative amplification expressed as percentage of control. Values represent the mean (± SD) of 3 independent experiments agents and confirmed the higher efficiency of BBR 3464 than CDDP in inducing DNA lesions. In fact, the concentration of BBR 3464 required to induce a 50% amplification inhibition was 14 times lower than that of CDDP (0.013 µM vs 0.183 µM).

The effect of DNA damage present in the N-*ras* gene of the 4 ovarian cancer cell lines was then evaluated immediately following a 5-h exposure of intact cells to CDDP or BBR 3464 and after an additional 6, 24 or 48 h in drug-free medium (Figure 2A). The experiments were carried out with the $IC_{50}$ of CDDP in all cell lines, whereas in the case of BBR 3464, a 10-fold $IC_{50}$ was used, since in A2780, A2780cp8 and OAW42MER cells the $IC_{50}$ values of the trinuclear platinum compound were too low to produce a level of DNA lesions quantifiable by the PCR technique. In OAW42 cells, a strong inhibition of signal amplification was observed at the end of CDDP exposure. The level of inhibition remained almost constant until 24 h after treatment, whereas at 48 h the extent of amplification increased to 67% of control, thus indicating a removal of drug induced DNA damage. Similar results were obtained after exposure of cells to BBR 3464, although in this case the extent of amplification after 48 h of recovery was significantly ($P < 0.05$) lower than that observed for CDDP and limited to 33% of control (Figure 2B). In CDDP-treated OAW42MER cells, an almost complete inhibition of signal amplification was detected 6 h after treatment. DNA damage was partially repaired at 24 h, and only very few lesions were still present at 48 h, as indicated by the extent of amplification, which was approximately 100% of control. Similar kinetics of DNA lesion accumulation and removal were observed after exposure of the cells to BBR 3464 (Figure 2B).

In A2780 cells, the maximum inhibition of signal amplification was found at the end of CDDP exposure. Progressive removal of DNA lesions, with a consequent increase in the extent of amplification, was observed starting at 6 h after treatment. At 24 h, the level of amplification reached 60% of control, and no additional increase was observed at 48 h. After exposure of the cells to BBR 3464, a negligible inhibition of signal amplification was observed up to 6 h, thus suggesting a slow conversion of monoadducts to bifunctional adducts. In fact, although theoretically Taq DNA polymerase is affected by both types of adduct, the PCR stop assay is expected to reflect mainly bifunctional adducts and in particular intrastrand cross-links, which represent the majority of drug adducts for CDDP (Murray et al, 1992; Bubley et al, 1994) and probably for BBR 3464 as well (Brabec et al, 1999). The highest level of amplification inhibition was detected at 24 h and it was almost unmodified at 48 h (Figure 2B). In A2780cp8 cells, the extent and the kinetics of accumulation and repair of CDDP-induced DNA lesions were similar to those observed with the same drug in A2780 cells, even though in A2780cp8 cells the highest inhibition of signal amplification (which should indicate the presence of the greatest number of lesions) was detected 6 h from the end of treatment. A slow kinetics of DNA lesion induction by BBR 3464 was recorded, and the maximum inhibition of signal amplification was detected 24 h after treatment. In these cells, which showed a moderate degree of resistance to BBR 3464, a slightly but significantly ($P < 0.05$) greater increase in the level of amplification, with respect to that observed in A2780 cells, was appreciable at 48 h, thus suggesting the ability of the cells to partially remove DNA damage (Figure 2B).

The results suggest possible differences in the kinetics of induction and removal of DNA damage between BBR 3464 and CDDP that may influence the cellular response to the 2 agents. Moreover, preliminary evidence suggests that the peculiar sensitivity to CDDP and BBR 3464 observed in our cellular models may also be attributable to a different ability to activate other pathways of cellular response to DNA damage, such as triggering of the apoptotic pathway, as a function of the genetic background of the tumour model (Orlandi et al, 2001).

### REFERENCES

Behrens BC, Hamilton TC, Masuda H, Grotzinger KR, Whang-Peng J, Louie KG, Knutsen T, McKoy WM, Young RC and Ozols RF (1987) Characterization of a cis-diammine-dichloroplatinum(II)-resistant human ovarian cancer cell line and its use in evaluation of platinum analogues. *Cancer Res* **47**: 414–418

Brabec V, Kasparkova J, Vrana O, Novakova O, Cox JW, Qu Y and Farrell N (1999) DNA modifications by a novel bifunctional trinuclear platinum phase I anticancer agent. *Biochemistry* **38**: 6781–6790

Bramson J and Panasci LC (1993) Effect of ERCC1 overexpression on sensitivity of Chinese hamster ovary cells to DNA damaging agents. *Cancer Res* **53**: 3237–3240

Britten RA, Warenius HM, White R, Browning PG and Green JA (1990) Melphalan resistant human ovarian tumour cells are cross- resistant to photons, but not to high LET neutrons. *Radiother Oncol* **18**: 357–363

Dignam JD, Lebovitz RM and Roeder RG (1983) Accurate transcription initiation by RNA polymerase II in a soluble extract from isolated mammalian nuclei. *Nucleic Acids Res* **11**: 1475–1489

Koberle B, Payne J, Grimaldi KA, Hartley JA and Masters JRW (1996) DNA repair in cisplatin-sensitive and resistant human cell lines measured in specific genes by quantitative polymerase chain reaction. *Biochem Pharmacol* **52**: 1729–1734

Manzotti C, Pratesi G, Menta E, Di Domenico R, Cavalletti E, Fiebig HH, Kelland LR, Farrell N, Polizzi D, Supino R, Pezzoni G and Zunino F (2000) BBR 3464: a novel triplatinum complex exhibiting a preclinical profile of antitumor efficacy different from cisplatin. *Clin Cancer Res* **6**: 2626–2634

Murray V, Motyka H, England PR, Wickham G, Lee HH, Denny WA and McFadyen WD (1992) An investigation of the sequence-specific interaction of cis-diamminedichloroplatinum (II) and four analogues, including two acridine-tethered complexes, with DNA inside human cells. *Biochemistry* **31**: 11812–11817

*© 2001 Cancer Research Campaign*

Orlandi L, Colella G, Bearzatto A, Abolafio G, Manzotti C, Daidone MG and Zaffaroni N (2001) Effects of a novel trinuclear platinum complex in cisplatin-sensitive and cisplatin-resistant human ovarian cancer cell lines: interference with cell cycle progression and induction of apoptosis. *Eur J Cancer* **37**: 649–659

Perego P, Caserini C, Gatti L, Carenini N, Romanelli S, Supino R, Colangelo D, Viano I, Leone R, Spinelli S, Pezzoni G, Manzotti C, Farrell N and Zunino F (1999) A novel trinuclear platinum complex overcomes cisplatin resistance in an osteosarcoma cell system. *Mol Pharmacol* **55**: 528–534

Pratesi G, Perego P, Polizzi D, Righetti SC, Supino R, Caserini C, Manzotti C, Giuliani FC, Pezzoni G, Tognella S, Spinelli S, Farrell N and Zunino F (1999) A novel charged trinuclear platinum complex effective against cisplatin-resistant tumours: hypersensitivity of p53-mutant human tumour xenografts. *Br J Cancer* **80**: 1912–1919

*© 2001 Cancer Research Campaign*

